# Evaluating the role of banking efficiency, institutions and financial development for sustainable development: Implications for Belt and Road Initiative (BRI)

**DOI:** 10.1371/journal.pone.0290780

**Published:** 2023-10-12

**Authors:** Wasi Ul Hassan Shah, Bo Wang, Rizwana Yasmeen

**Affiliations:** 1 School of Management, Zhejiang Shuren University, Hangzhou, China; 2 Department of Economics, University of Religions and Denominations, Qom, Iran; 3 International Business School, Southwestern University of Finance and Economics, Chengdu, China; 4 School of Economics and Management, Panzhihua University, Panzhihua, Sichuan, China; Shenzhen University, CHINA

## Abstract

This study explores the relationship between banking efficiency and financial development in the Belt and Road Initiative (BRI) economies from 2007 to 2018. The study employs three dimensions to assess financial development: (i) depth, (ii) stability, and (iii) efficiency. In the initial stage, BRI banking efficiency is quantified using Data Envelopment Analysis (DEA). Subsequently, the Generalized Method of Moments technique is applied to identify the connection between banking efficiency and financial development. The study employs fundamental structural benchmarks to evaluate disparities between actual financial development indicators and predicted values. Banking efficiency plays a crucial role in determining the depth, stability, and efficiency of financial development within BRI economies and is pivotal in closing these gaps. Strong institutional frameworks also support the advancement of the BRI’s financial development sector. Moreover, foreign direct investment positively impacts reducing financial development gaps and promoting growth in the financial sector. The study concludes that BRI member countries should prioritize banking industry reforms to enhance the stability, depth, and efficiency of their financial sectors.

## 1. Introduction

The Belt and Road Initiative (BRI) is a historical economic initiative of the Chinese government covering 65 countries worldwide [1–3]. The sole convincing force behind this initiative is to promote sustainable economic development through collaborations from the Western Pacific to the Baltic Sea. The demand for capital has been increasing for different projects since the initiation and practical execution of the BRI. Infrastructure development is at the core of the BRI economic initiative to accelerate economic activities and optimize resource allocation [4]. Additionally, under the economic vision, the BRI invests in energy development projects and free trade economic zones. As a result, an effective financial system is essential to support the sustainable development of the BRI economic initiative. BRI offers many opportunities for investors to invest in different business sectors. Therefore, the role of financial entities, such as banks, cannot be ignored. Financial institutions facilitate investors in providing credits and approval procedures via modern technologies [5, 6]. They are intermediaries between the depositor and borrower to maximize their utility and profit through efficient operational strategies [4].

Meanwhile, BRI promoted banking services through the Asian Infrastructure Investment Bank and Silk Road Fund. The BRI countries, about 57, have signed the articles of agreement of the Asian Infrastructure Investment Bank for official banking services [7]. In 2017, the Deutsche Bank and China Development Bank signed a memorandum of understanding to support global investors in renewable energy sectors, infrastructure, and commodities, corroborating as the primary financial vehicle to support BRI projects. Besides, Beijing has invested much capital into the Chinese Export-Import Bank and the Chinese Development Bank with low borrowing costs to provide quick and cheap access to loans for Chinese companies involved in BRI projects. Banks are vital to supporting the current financial system; however, their efficiency is essential to economic development [8]. An inefficient banking system with high lending interest rates can increase lending risk, reflecting currency and other macroeconomic uncertainties.

Similarly, efficient and productive banks expand businesses, maximize profit in the competitive market, and allow competitors and regulatory authorities to get maximum output from available resources [9]. However, ownership, operating location, and size significantly affect the efficiency and productivity of banking all around the globe [10]. Thus, banking efficiency is another factor in the financial sector that supports the sustainable development of the BRI economic initiative by motivating savers to invest in profitable projects.

Additionally, the quality of the financial sector in terms of banks and financial development is coupled with institutions that would lessen the financial risk and transaction costs for finance seekers [11]. The institutions such as government, financial, and property rights warrant safe and sound legal protection for investors and shareholders in financing projects [12]. Numerous studies have acknowledged property rights as building blocks in sustainable development and industrialization [13, 14]. A well-defined property rights system stimulates capital investment and expedites technology transfer. Property rights enforce institutions to ensure solid legal protection for investors in financing projects for the sustainable development of the BRI economic initiative. Property rights are a stimulating factor for the stakeholders in financial projects. On the other hand, financial liberalization improves the efficiency of banks as banks enjoy liberalization in the provision of their services.

A higher degree of liberalization assists the banks in increasing cost efficiency and providing low-priced loans to clients [15]. Capital liberalization facilitates and enhances the domestic financial system’s efficiency as it helps to allocate the saving to the most productive usage [16]. However, due to country-specific characteristics, financial liberalization may not always be efficient in all economies. Furthermore, the effects of financial liberalization depend on the country’s financial markets and the quality of institutions. Additionally, government integrity can play an influential role in controlling state departments by implementing rules and regulations. In the same vein, China’s government integrity has become increasingly crucial to the sustainable development of BRI. Since investors only will invest if the government keeps its word and promises [17]. High-level government integrity leads to fair practices between the government and the enterprises [18].

Moreover, it has been witnessed that BRI is a way forward for China to increase its economic power as a creditor. Therefore, the financial sector’s development and the institutions’ quality are increasingly essential to BRI’s sustainable growth. Furthermore, a country’s financial development level is determined through structural characteristics (Growth, demographics, age dependency, population, and other fundamentals) outside policy controls [19]. Considering benchmarks are calculated using structural variables. Structural variables identify the differences between the expected benchmarks and actual financial development values. The gaps between the projected standards and actual financial development values are defined to measure the financial sector gap. Based on the analysis, it is argued that persistent overshooting of the benchmark is related to the possibility of a bad credit boom and loans.

These arguments support our study and motivate us to fill this vacuum that hasn’t been extensively covered. In this regard, the following aspects are the novel contributions of the present study. Firstly, the technical banking efficiency is gauged by applying Data Enviplment analysis for BRI countries. To the authors’ knowledge, it has not been performed before for the BRI region. These efficiency scores are essential for the second stage’s empirical exercise. Secondly, in the next stage of the estimation, the GMM approach is applied to unveil the bonding between banking efficiency and financial development. Third, Using a single metric to measure financial development is inadequate due to the financial system’s diversity. Therefore, three dimensions of financial systems are employed: (i) financial development depth, (ii) financial development stability, and (iii) financial development efficiency. It would add a freshness to the canon of previous works. Fourth, the benchmarks for the panel of BRIs countries to define the financial gaps (e.g., depth, stability, and efficiency gaps) are analyzed. At Fifth, we incorporated the role of financial institutions that are more important for the financial sector: (i) financial liberalization, (ii) property rights, and (iii) government integrity. Lastly, we also raise the importance of foreign direct investment, which often gets overlooked when evaluating financial development. The rest of the study is organized as follows: Section 2 describes the detailed literature review. Sections 3 and 4 present the theoretical framework and materials and methods used in the research. Data sources and results’ discussions are described in sections 5 and 6, respectively. The conclusion of the study is described in the last section.

## 2. Literature review

Financial sustainability is central to an economy aimed at fostering sustainable development. Furthermore, the finical sector certainly affects the growth of the economies. Among researchers, Schumpeter [20] linked financial development with growth in his pioneer work, identifying the banks’ importance in supporting innovations and development. Efficient banks benefit investors and the entire economy [21]. Furthermore, the banking sector is important in developing the financial sector, which empowers the environment with constant growth. Therefore, the efficiency of banks has been the hub of academic researchers and policymakers. The existing literature on the role of banking efficiency and economic institutions on financial development is discussed in the following sub-sections.

### 2.1 Banking efficiency and financial development

The first strand of literature focused on banks and their efficiency. The selection of the inputs, output variables, and methods of estimations are different in various studies. In some studies, the Data Envelopment Analysis approach is applied to measure the productivity and efficiency of the banking industries. Hsiao et al. [22] carried out the DEA approach with three inputs (interest expenses, non-interest expenses, and total deposits) and three outputs (interest revenue, non-interest revenue, and total loans) to quantify the operating efficiency in Taiwan. They found lower operational efficiency during the reform periods (2002–2003) compared to the pre-reform period (2000–2001). However, there was significant operational efficiency in the post-reform period. Kablan [23] investigated banking efficiency to elucidate the level of financial development in sub-Saharan Africa. In the first part of the study, the study applied the stochastic frontier analysis with the one-step method to calculate the banking efficiency and found that sub-Saharan African banks are cost-efficient. In the second stage, he used the GMM method and concluded that banking efficiency could be increased under a well-regulated, sound, rigorous credit system. Karim et al. [24] applied a semi-non-parametric Fourier flexible, functional approach and concluded that African banks would become more cost-efficient if they operated efficiently.

Additionally, the study found a bi-direction causality between financial development and banking efficiency. In contrast, Fernandes et al. [16] applied DEA using the Malmquist Productivity Index to determine the efficiency of peripheral European domestic banks. Furthermore, compared to Yobe [25], this study used truncated regression to inspect whether changes in the financial conditions affect differently to the level of banking efficiency. Their analysis found that credit risk and liquidity hurt banks’ productivity. Nevertheless, capital and profit risk are positive in parallel to banking performance. Royo et al. [26] used different measures, such as size and the efficiency of intermediaries and markets, concluding that banking stability is enriched in market-based financial systems. Further literature certified that banking efficiency is essential to enhance the financial sector’s ability by reducing risk and cost.

### 2.2 Economic institutions and financial development

The second strand of literature is about the institutional determinants of financial development. The study by Berger et al. [27] found that the solid institutional framework effectively works in developing equity and credit markets. Similarly, by applying OLS methods, Breuer [28] concluded that there is a positive association between capital allocations and the legal protection of minority investors. Using a panel approach, Chen et al. [29] highlighted the significance of institutions for credit cycles. They learned that improvements in operative creditor rights led to a decrease in the volatility of the credit cycle. Further, advancing effective creditor rights helps keep the credit market’s dimensions and scope.

#### 2.2.1 Property rights

Certain empirical studies pointed out that institutions ensuring strong property rights were thought to spur financial markets. Such as selecting a data set for 20 OECD countries on a sample of 22 manufacturing industries from 1990 to 2009 Maskus et al. [30] analyzed the combined impact of domestic and international financial market development and patent protection on R&D intensities. The results have shown that the effects of patent protection on R&D investment are imperative through bank lending and external equity. Additionally, they suggested that bond financing was beneficial for allocating innovative capital wherever financial markets are already efficient and allowing more arm’s length transactions. Claessens et al. [31] conducted an empirical study using extensive financial indicators such as financial development, property rights, and growth channel. They argued that a country with weak property rights and poorly developed financial systems has two significant leading effects on firms. Firstly, it is a big hurdle for firms to approach outdoor financing. Secondly, firms could not use their resources optimally. The study of Yoo and Steckel [32] on Property Rights and Financial Development in the Legacy of Japanese Colonial Institutions showed that well property-defining institutions stayed essential for economic development. Miletkov and Wintoki [33], through a panel study of 129 countries, tested the role of financial development in the evolution of property rights and legal institutions. The findings revealed a beneficial causal relationship between the institution of property rights and the degree of financial development. The Middle East and North African nations have also been shown to have favorable interactions [34, 35].

#### 2.2.2 Financial liberalization

Since the 1970s, countries gradually started liberalizing their financial systems by lowering interest rates and credit controls and opening capital accounts. World Bank [36]. believe that financial institutions can minimize market imperfections and allocate capital to its most productive use. Demirgüç et al. [37] suggested that the quality of institutions is essential for successful financial liberalization. They stated that the rule of law and law enforcement are important, as the absence of proper regulations and financial liberalization increased the probability of banking crises. According to Suryanto et al. [38], financial liberalization- using restrictions on deposit, lending interest rates, and reserve and liquidity requirements- towards financial development is complex and varies substantially across the sample of countries. Yet, the variations in the results are driven by institutional differences, such as the lack of proper regulation and supervision. Zisko et al. [39] evaluated that capital account openness promotes financial deepness and enjoying high growth. These assessments were generally based on data from stable economies. At the same time, countries with poor institutional quality, bank regulations, and open capital accounts policies cannot support financial development. Elkhuizen et al. [40] also pointed out that the impact of financial liberalization policies on financial development differs significantly across countries due to the institutional environment. They argued that the quality of formal institutions is a vital factor in harnessing the benefits of financial liberalization. However, social capital may substitute for formal institutions. Countries with a high prevailing social capital level benefit from financial liberalization, regardless of the quality of their formal institutions. However, Jawad et al. [41] found the positive effects of capital account openness on financially-dependent industries. Accordingly, countries must first reach a particular institutional and economic development level to reap the benefits of capital account liberalization.

### 2.3 Government institutions

Governments with integrity must establish a sound social credit system and promote a conducive investment environment primarily through private capital investment. Simatupang [42] examined the impact of government size and quality on the financial sector, size, and efficiency of 71 economies. The study found evidence that the quality of the government–in terms of governance and legal origin- positively affects financial sector efficiency and size. However, the government size measured by government expenditure and the government ownership of banks negatively influence the financial sector but positively affect the size of the financial industry. At the same time, Porta et al. (1998) [43] determined that government ownership of a banking system negatively influences financial sector development and economic growth. However, Al-Matari et al. [3] emphasized the importance of institutions for financial sector development. The study argued that the government could help banks to start economies with poor institutional quality.

Further, they argued that governments should encourage private-sector banking. Al-Matari et al. [3] suggested that institutional factors such as deposit contract enforcement are more important determinants of the share of state banks than political ones. Further, they argued that governments should build institutions that promote the private banking system, particularly in developing countries. By employing the banking sector and stock market development measures, law et al. [44] explored that institutional quality, such as the rule of law, government effectiveness, and political stability, are sources of financial development. Studies examined the impact of institutions on the financial development of 52 developing economies. They demonstrated that international differences in the level of banking sector development are driven by institutional quality. However, joint reforms of institutions matter significantly compared to separate institutional reforms [45].

## 3. Theoretical framework

Financial development occurs when financial intermediaries such as banks, insurance companies, stock markets, and bond markets remove the market frictions that restrain the free flow of financial assets from money holders to borrowers and help diversify risks [46]. It mobilizes saving, which is a cornerstone of long-term economic growth. Although there is substantial literature on the role of financial development in shaping economic development, most researchers consider one dimension of financial development, i.e., the banking industry’s size. Considering one dimension might not be enough to assess the entire performance of the financial development [36]. The following three measures were used to compare the multidimensional nature of financial systems: (i) financial development depth, (ii) financial development stability, and (iii) financial development efficiency. Each indicator is associated with financial sector policies and is strongly related to socio-economic development that enables an environment for finance. For example, credit to GDP is used to measure the financial development depth [11, 47]. It reveals the degree to which people have the financial opportunity provided by financial entities. In line with Almarzoqi [46], non-performing loans are peroxided to assess the stability of financial systems. In addition, how effective and efficient the financial system is in terms of the financing environment is calculated by the financial entities’ profitability (e.g., returns on assets) [36, 48].

Besides, the banking sector is an integral part of the financial systems. Yet, the efficiency of financial intermediaries like more conventional banks can mitigate macroeconomic uncertainties. Therefore, the second central tenet of this research work is to gauge the behavior of banking efficiency toward financial development. The technical efficiency of BRI has been calculated by employing the DEA technique (Banker et al. 1984) [49]. The economic institutions are the appealing factors that promote sustainable financial system development and provide space for productive market-based economies [50, 51]. This study uses three institutions that are more relevant to the financial sector to bring about stability in BRI. Among the three, property rights are the main convincing factor for investors to engage their financial resources in BRI projects [11, 47]. Correspondingly, banks would facilitate investors efficiently if they were financially liberalized and had the freedom to serve their customers. Government can protect the people’s interests and fulfill their promises by implementing the rules and regulations [18]. Additionally, local governments can open the door to greet the investors; hence, government integrity is the basic element to ensure that the enterprises benefit from the investment. The present study highlights the role of institutions, namely property rights, financial freedom, and government integrity, in acquiring financial development for BRI countries.

**Fig 1:**
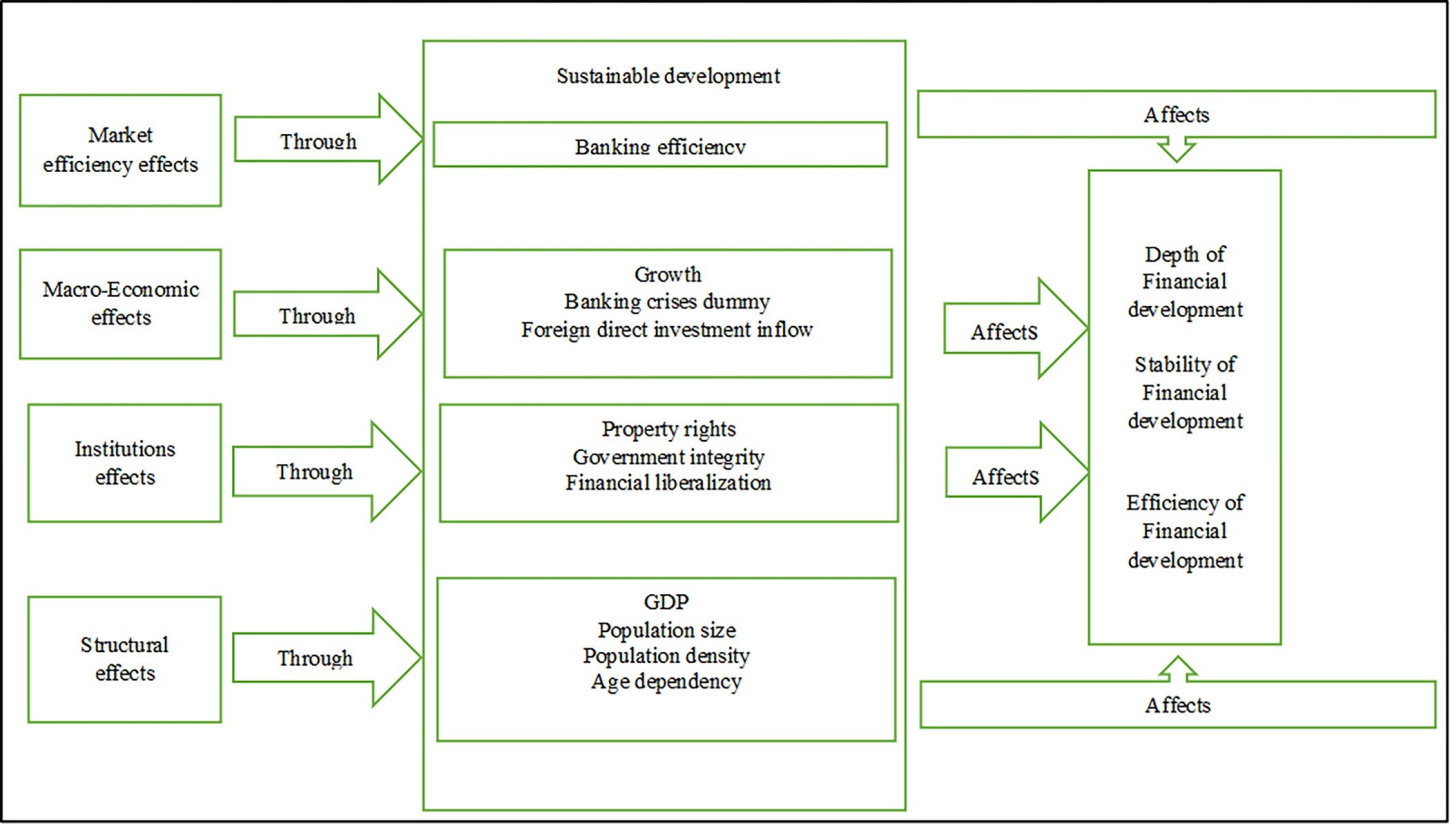
Analytical framework for financial development across different dimensions.

Based on the literature, the analytical framework of financial development is elaborated in Fig 1.

Vis-à-vis this mechanism, we compose the functional form Barajas and Kablan [13, 19].


$$Y_{it}=Y_{i,t-1}+{\alpha_{i}P}_{it}+\beta_{i}{{ST}_{it}+u}_{it}$$ (1)

Where, *Y*_*it*_ represents the financial development depth, stability, and efficiency for country i and year t. *P* is vector variables described in Fig 1, such as the banking efficiency of BRI economies (BE), property rights (PR), the degree of financial liberalization (FL), government integrity (GI), country growth (GDP), the inflow of foreign direct investment (FDI), and banking crises of BRI countries (BCR_dummy). In contrast, ST is the vector of structural variables (population, age dependency, and population density).

In the line, Barajas et al. [19] calculate the benchmark for the panel of BRI countries to define the financial gap (i.e., the difference between the actual level of financial development and the level predicted by structural characteristics). Following the benchmark analysis, the gap analysis used key structural variables argued by Demirgüç-Kunt et al. [52] and shown in Fig 1. Financial development is affected by economic growth as the level of income increases and the demand for financial intermediation also rises. Demographic changes are also significant as these can affect the financial markets services and the need for financial assets [53]. Additionally, a growing population and population density contribute to a greater potential demand for intermediation, which further reinforces the network impact of delivering financial services. Age dependence and loaning disposition are both considered to be good saving factors.

The following dynamic model is used for the gap analysis:

$${Y\_GAP}_{it}={Y\_GAP}_{i,t-1}+\alpha_{i}P_{it}{+u}_{it}$$ (2)

Here, *Y*_*GAP*_*it*_ represents the deviation from the structural benchmark of a specific dimension of financial development. *P* is the vector of variables outlined in the analytical Fig 1, such as the banking efficiency of BRI economies (BR), property rights (PR), the degree of financial liberalization (FL), government integrity (GI), country growth (GDP), the inflow of foreign direct investment (FDI), and banking crises of BRI countries (BCR_dummy).

## 4. Materials and methods

This study first employed Data Envelopment Analysis (DEA) to measure the banking efficiencies and then applied the Generalized Moment Method (GMM) to empirically estimate factors of financial development.

### 4.1 Data envelopment analysis

To measure the efficiency of Decision-Making Units (DMUs), Charnes et al. [54] introduced a nonparametric analysis known as (CCR) DEA. This linear programming technique was initially used to measure the technical efficiency of DMUs. In their pioneering work, Sherman et al. [55] applied DEA to the banking sector to calculate the relative efficiency scores of various DMUs. Numerous studies employed DEA to evaluate banking efficiency in different countries [56–58].

### 4.2 CCR model

Considering a set of *J* DMUs with *n* input and *m* output in *T* (*t* = 1,…, *T*) periods. Suppose in time *t*, decision-makers are using inputs $x^{t}\in R_{+}^{n}$ to produce outputs $y^{t}\in R_{+}^{m}$. Define the input requirement set in period *t*, which is:

$$L^{t}(y^{t})=\{x^{t}:x^{t}can\;produce\;y^{t}\}$$ (3)

Assume that *L*^*t*^(*y*^*t*^) is non-empty, closed, convex, bounded, and satisfies the strong disability property of inputs and outputs *L*^*t*^(*y*^*t*^) determined from below by the input isoquant (a constant returns to scale (CRS) production boundary) that is:

$$\mathrm{Isoq}L_{}^{t}(y^{t})=\{x^{t}:x^{t}\in L^{t}(y^{t}),\;\lambda x^{t}\notin L^{t}(y^{t})\;\mathrm{for}\;\lambda <1\}.$$ (4)

Define the input distance function of period *t* as follows:

$$D_{}^{t}(y^{t},x^{t})=\underset{\theta }{\sup}\{\theta :(x^{t}/\theta )\in L^{t}(y^{t}),\theta >0\}.$$

Hence, the DEA-CCR model for measuring TE in period *t is* as follows:

$${\mathrm{TE}}^{t}(y^{t},x^{t})=1/D_{}^{t}(y^{t},x^{t}).$$ (5)

Usually, TE<1 indicates that a specific DMU is under assessment compared with other DMUs. It shows that this DMU is productively inefficient because it used excessive inputs, while TE = 1 shows that DMU is fully efficient.

### 4.3 Panel Generalized Methods of Moments (GMM)

Cross-section studies mainly involve endogeneity issues. The lagged dependent variable on the right-hand side leads to correlation problems with the unobserved country-specific effects. In this case, OLS and fixed effect methods are unsuitable and give biased and inconsistent results [51, 59]. The GMM is the most popular strategy if the period is less than cross-sections, as it erases the fixed effects via transforming the first difference [60]. Due to the reasons above, we applied GMM’s first difference. GMM’s first difference is also used to mitigate the unobserved country-specific effect. Omitted indicators and endogeneity problems via instrumenting the explanatory regressors.

To avoid the correlations between the error terms and lagged dependent variables, we used lagged values of the explanatory variables as instruments [51]. Moreover, we employed the Arellano-Bond test AR (1) and AR (2) to remove serial correlation in the residuals. Here we have reported AR (2) for second-order autocorrelation for each model.

## 5. Data sources

**Fig 2:**
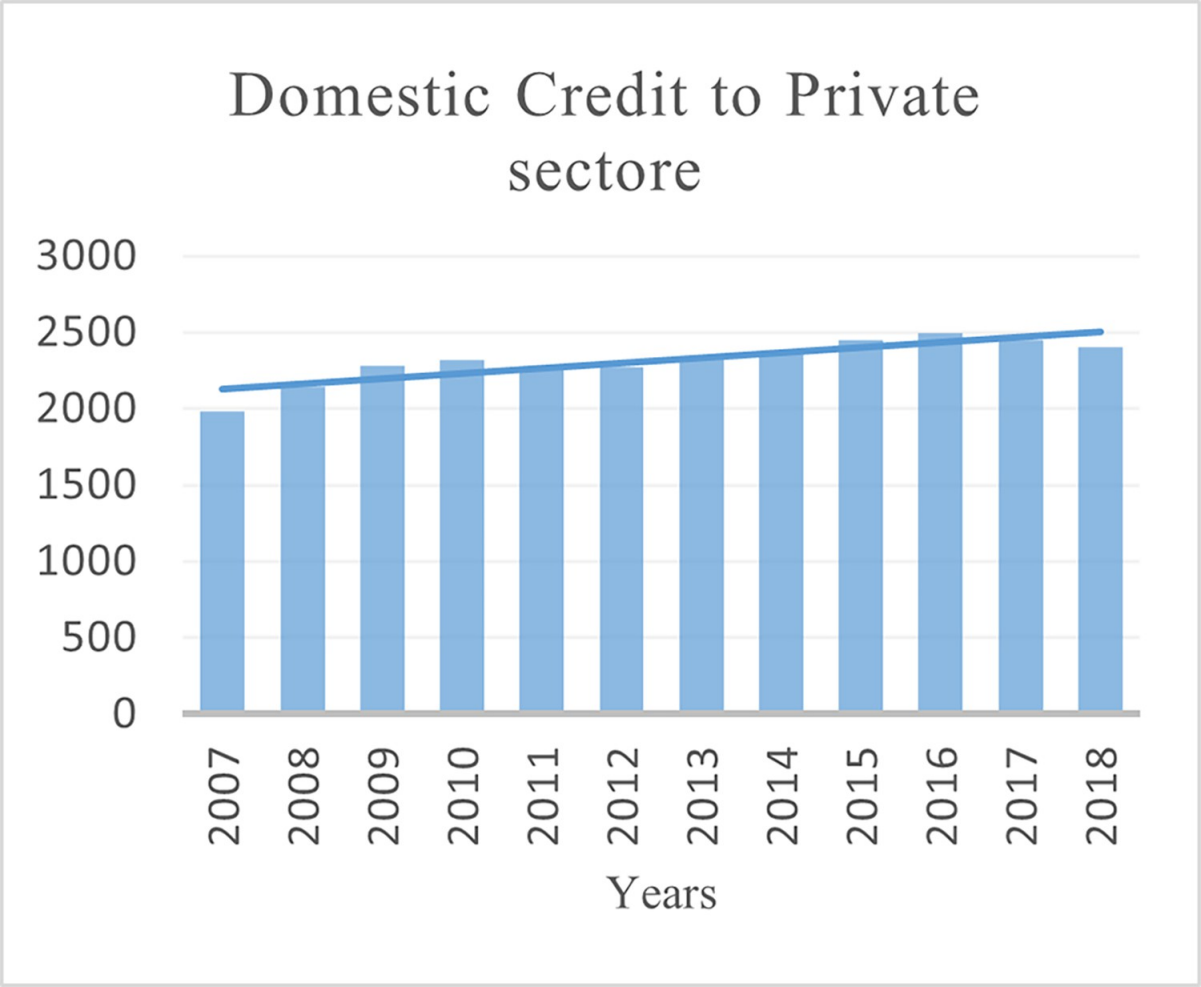
Domestic credit of BRI economies banks.

**Fig 3:**
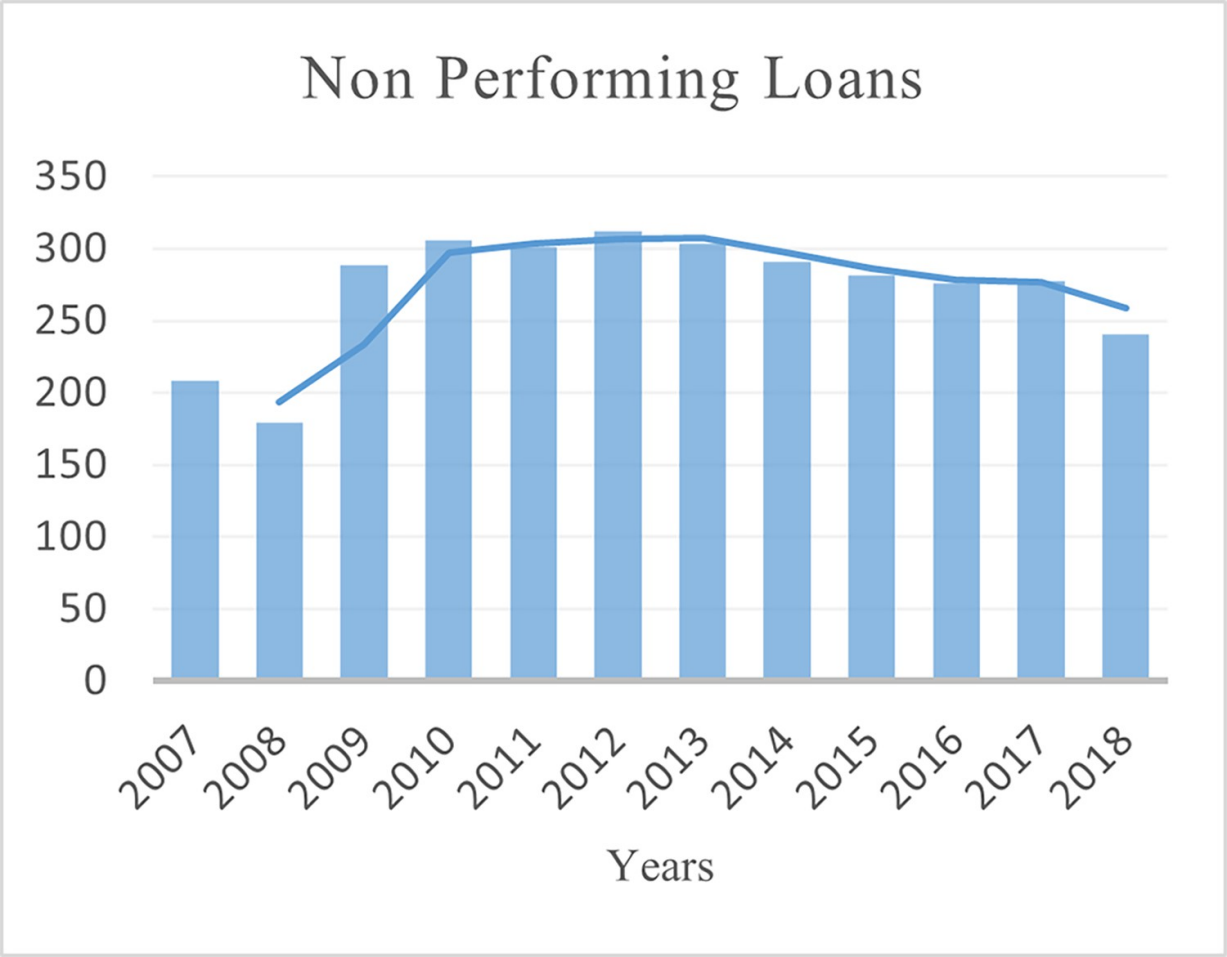
Non-performing loans of BRI economies banks.

**Fig 4:**
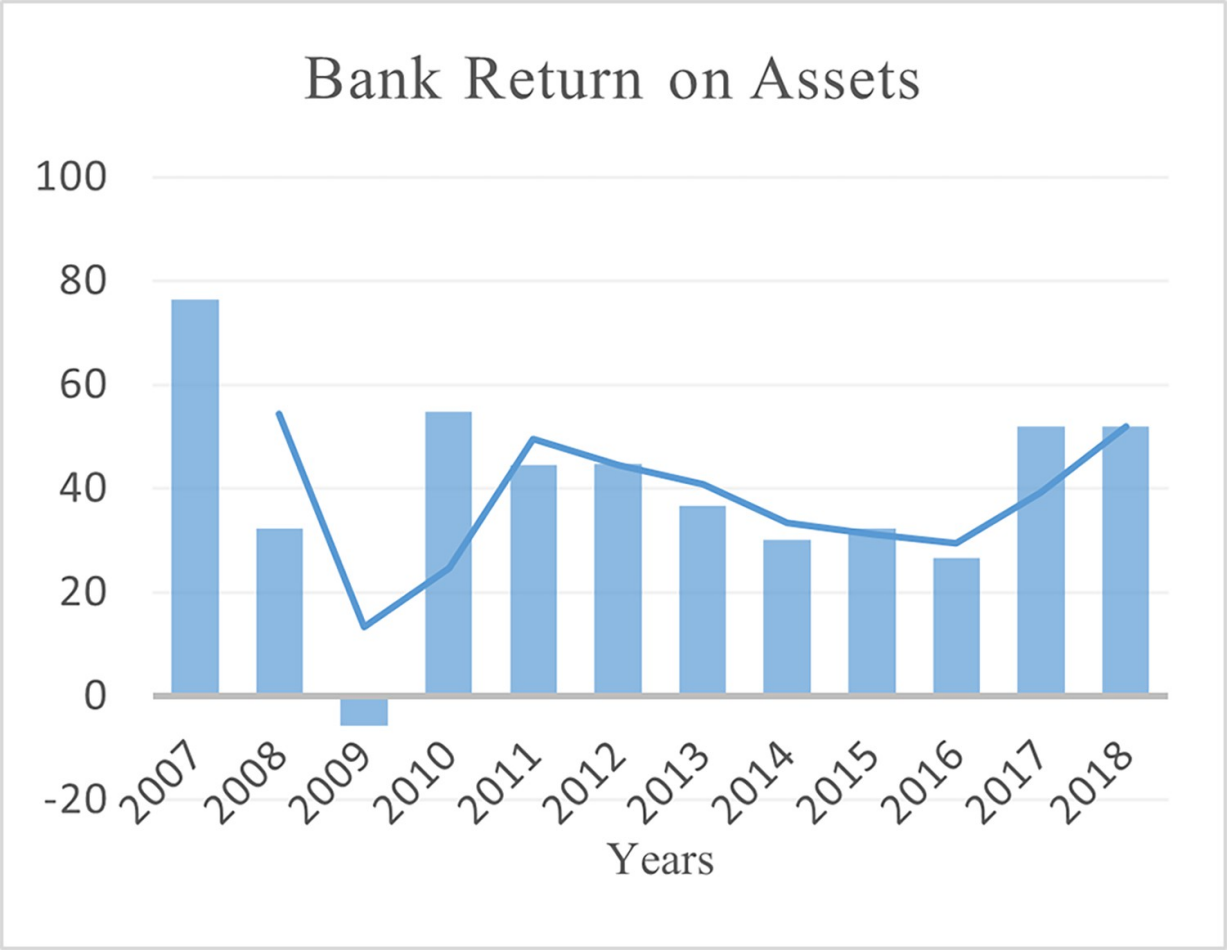
Bank returns on assets of BRI economies.

This paper uses the balance data of 40 BRI regions (see Table A1 in S1 Appendix) for empirical analysis (2007–2018). The selection of the countries and period for the study is based on the availability of the data. The three dimensions of financial development are measured as domestic credit to the private sector to GDP ratio for depth (see Fig 2). Non-performing loans as a percentage of total gross loans for stability (see Fig 3) and Bank return on assets for efficiency (see Fig 4). Financial development data is collected from the World Bank’s Global Financial Development (GFDD) database.

**Fig 5:**
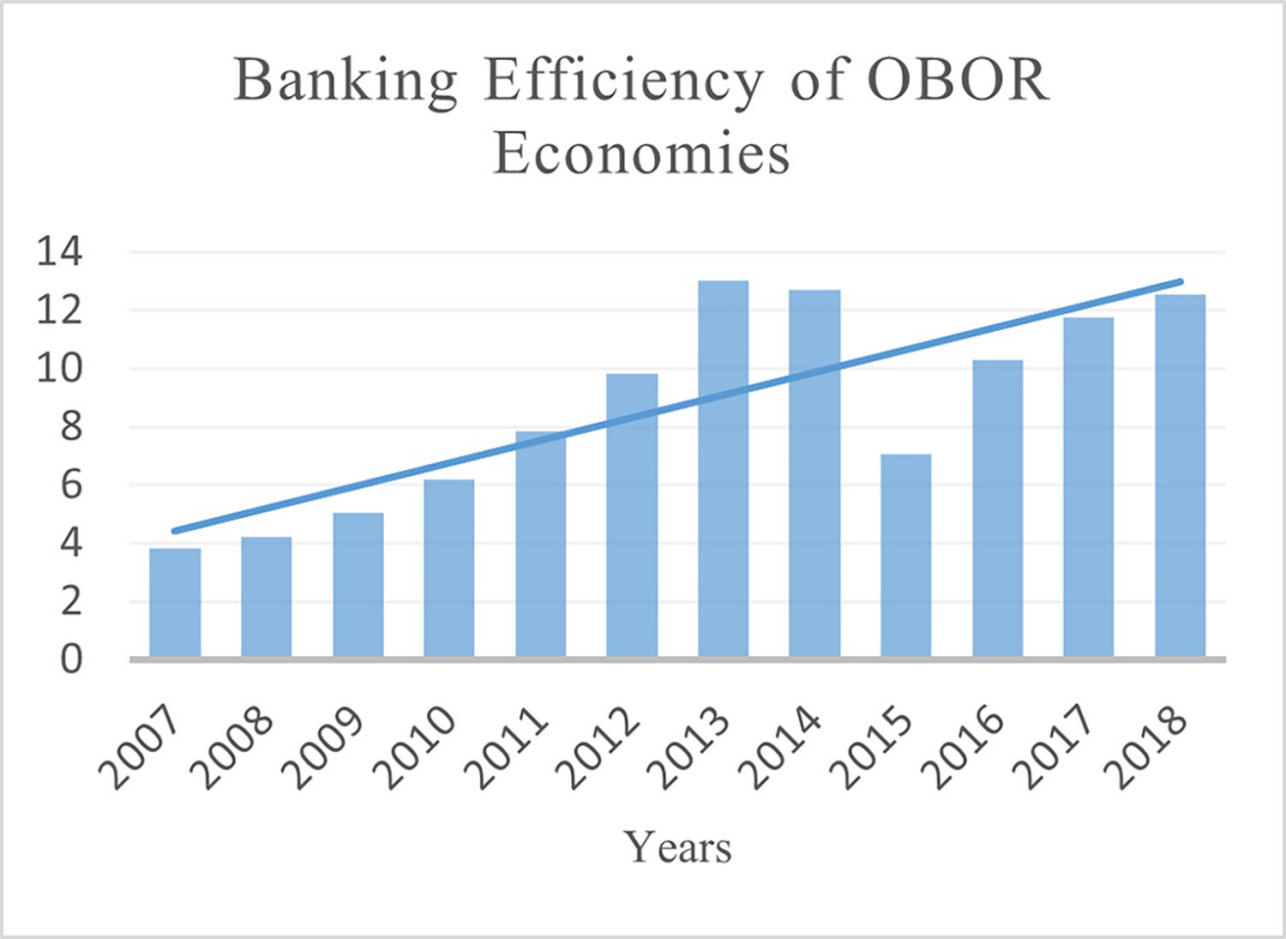
Banking efficiency of BRI economies.

We used DEA-CCR (input-oriented model) to estimate the banking efficiency. With two inputs (interest expense, non-interest expense) and two outputs (interest income, non-interest income) were employed to measure the technical efficiency for each DMU of every country in the sample (see Fig 5). Separate efficiency evaluations were conducted for each BRI country to tackle the production technology heterogeneity between their respective banking sectors. The dataset of 1272 banks included all commercial banks available for a fiscal year in the specific banking industry of BRI countries. Input-output variable data was collected through the bank focus database while missing values were collected for some banks through the annual reports of a specific country’s central banks. The data of other macroeconomic variables (foreign direct inflow, GDP per capita, and the dummy for banking crises) are obtained from the World Development Indicators and Global Financial Development (GFDD) database. The data for economic institutions, property rights, government integrity, and financial freedom indicators are obtained from the Index of Economic Freedom (2018) published by the Heritage Foundation Index of Economic Freedom indicators range from 0 to 100. For a detailed description of the data (see Table 1).

**Table 1:** Variable description.

Variables	Notation	Quantification	Data source
**Financial Development variables**			
Domestic credit to the private sector	FD Depth (PC)	Domestic credit provided to the private sector by financial corporations, monetary authorities, deposit money banks, as well as other financial corporations (% of GDP)	GFDD
Non-Performing Loans	FD Stability (NPLS)	Non-performing loans to total gross loans	GFDD
Returns on assets	FD Efficiency (ROA)	Bank returns on assets (%)	GFDD
FD Depth Gap	FD Depth Gap (PC)	The difference between the actual value of domestic credit to the private sector and the predicted benchmark value which is divided by the benchmark value. Benchmarks are calculated by using regressions of indicators on structural indicators across BRI’s panel. *GAP*_*it*_ = (*level*_*it*_−*benchmark*_*it*_)/*benchmark*_*it*_	GFDD,
FD Stability Gap	FD Stability Gap (NPLS)	The difference between the predicted benchmark value and the actual value of non-performing loans divided by the benchmark value ${GAP}_{it}=\frac{{benchmark}_{it}-{level}_{it}}{{benchmark}_{it}}$ (In this, we reverse the sign)	GFDD
FD Efficiency Gap	FD Efficiency Gap (ROA)	The difference between the benchmark value and the returns on assets divided by the benchmark value ${GAP}_{it}=\frac{{benchmark}_{it}-{level}_{it}}{{benchmark}_{it}}$ (In this, we revers the sign)	GFDD
**Market Efficiency**			
Banking Efficiency (technical efficiency)	BE	Two inputs (interest expenses and non-interest expenses) and two outputs (interest income and non-interest income	Bank focus database and Annual reports of central banks of the particular country
**Institutional variables**			
Property rights	PR	To the extent a country’s Private property is protected by the government (index)	Index of Economic Freedom
Financial freedom	FL	The degree of liberation of financial or capital institutions and the extent they are free to provide finance. (index)	Index of Economic Freedom
Government Integrity	GI	Transparent system in which a country is free from corruption and has an effective administrative system (Index)	Index of Economic Freedom
**Macroeconomic variables**			
Growth	GDP	GDP per capita (constant 2010 US$)	World Development Indicators
Foreign direct investment	FDI	Foreign direct investment, net inflows (% of GDP)	World Development Indicators
Banking Crisis	BCR_ dummy	Dummy indicator used for banking crisis (1 = there is a banking crisis, 0 = there is no banking crisis).	GFDD
**Structural Variables**			
GDP per capita	GDP	GDP per capita (constant 2010 US$)	World Development Indicators
Square of GDP per capita	SGDP	Square of GDP per capita (constant 2010 US$)	World Development Indicators
Age Dependency	Age _ den	Acquired by Adding the younger (people younger than 15) and older (older than 64) dependency ratio. (% of working-age population)	World Development Indicators
Population density	POP _ den	Population density is midyear population divided by land area in square kilometers (Population density (per sq. km of land area)	World Development Indicators
population	POP	Total population	World Development Indicators

The correlation matrices for financial development and financial development gap with specific dimensions are given in Tables A2 and A3 in S1 Appendix. The matrix shows that banking efficiency positively correlated with financial development depth and efficiency. It also indicates that banking efficiency increases financial development stability by lowering non-performing loans. In contrast, the gap correlation matrix shows a negative correlation between banking efficiency, financial depth, and financial efficiency gaps. However, the bonding between the stability gap and banking efficiency is positive. This correlation implies that banking efficiency promotes financial development by adopting technological innovations in the banking system, making finance easy to access. Economic institutions such as property rights, financial liberalization, and government integrity show that institutions can improve the ongoing financial structure and performance.

## 6. Results and discussion

First, the DEA-CCR input-oriented model with two inputs (interest and non-interest expenses) and two outputs (interest and non-interest incomes) is applied to measure the technical efficiency of the banking sector for BRI countries. The technical efficiency scores given in Table A4 in S1 Appendix showed that the technical efficiency of banks in the BRIs countries improved during the sample period. The p-value of the j-stat is more than 5 percent confirming that the instruments are valid in each regression.

Table 2 reports financial development depth, efficiency, and stability results. In column 1 of Table 2, we examine the determinants of financial development depth. The findings show that banking efficiency has a positive and significant relationship with financial development depth. A positive connection suggests that the financial system performs better with depth. Our results are consistent with Banyen [61], who concluded that banking efficiency increases financial development in sub-Saharan countries. However, banking crises negatively influence financial development depth. The institutional structure is always essential to regulate the financial system. The impact of government integrity and financial liberalization on financial depth is significant. It is also concluded that growth enhances financial depth. Increasing foreign direct investment in BRI countries affects financial depth positively if the institution devises policies wisely. The findings are aligned with [23, 62, 63].

**Table 2:** Financial development–analysis.

Variables	FD depth (PC) (lag-1)	FD stability (NPLS) (lag-1)	FD efficiency (ROA) (lag -1)	BE	PR	LR	GI	GDP	FDI	BCR_ dummy	POP	Age_dep	POP_dens
FD depth (PC)	0.389***			0.154***	0.0018	0.0073***	0.0028**	0.451***	0.0081***	-0.068**	0.229	1.217***	0.221
	(0.000)			(0.000)	(0.359)	(0.000)	(0.021)	(0.000)	(0.000)	(0.004)	(0.688)	(0.000)	(0.708)
FD stability (NPLS)		0.558***		-0.186***	-0.0034***	0.0066***	-0.0013***	-0.252***	-0.0040***	0.237***	-3.901***	0.067	-3.730**
		(0.000)		(0.000)	(0.000)	(0.000)	(0.000)	(0.000)	(0.000)	(0.000)	(0.000)	(0.310)	(0.000)
FD efficiency (ROA)			0.057***	2.365***	0.011***	0.0036***	0.0221***	1.654***	0.0022***	-0.413**	-7.170***	-3.058***	-4.469***
			(0.000)	(0.000)	(0.000)	(0.000)	(0.000)	(0.000)	(0.000)	(0.019)	(0.000)	(0.000)	(0.001)
j-stat	33.578	32.317	32.738										
Prob	(0.255)	(0.306)	(0.288)										
AR(2)	-1.255	-0.1934	0.066										
	(0.209)	(0.846)	(0.946)										

Note: ***, **, * show significance level at 1%, 5%, and 10% respectively. P-values are given in parentheses. J-stat prob. greater than 5% shows that instruments are valid.

The financial development stability and stability gap determinants are assessed with non-performing loans in the banking sector. In the case of an efficient financial sector, it would be rational to have a more stable financial development system. Therefore, banking efficiency negatively affects non-performing loans (Table 2). Higher efficiency of the banking sector would increase the actual returns on projects and amplify the capacity to pay back loans, decreasing the volume of non-performing loans. The impact of growth in column 2 of Table 2 indorses that higher growth increases the stability of the financial development sector by reducing non-performing loans. However, financial liberalization is detrimental to financial development stability. It implies that liberalization can destabilize the financial system by crediting non-performing loans while government integrity once again is significant to uplift the financial development stability. Property rights also seem to be an encouraging factor in stabilizing the financial system. Foreign direct investment results suggest that financial development is a good sign for raising financial development stability as project returns increase, thereby releasing the debt burden. It has been shown by the dummy crisis coefficient that banking crises decrease financial development stability. Structural variables show a negative impact on financial development stability. Cuadrado-Ballesteros [64] also argue that government integrity boosts financial development.

The dimension for the financial development efficiency is evaluated using returns on assets in the banking sector. The results in column 3 of Table 2 show that higher banking efficiency would imply higher asset returns. The coefficient of financial liberalization in column 3 of Table 2 is positive and highly significant. It implies that the degree of freedom plays a vigor role in promoting financial development efficiency and, thus, growth in BRI economies. The government’s integrity role seems supportive of promoting financial development efficiency. Such as, the coefficient impact of government integrity on financial development efficiency is positive in column 3 of Table 2. Also, the findings in column 3 of Table 2 certified that property rights help to develop financial efficiency. The policy variables, growth, and FDI also improve financial institutions’ efficiency. The negative impact of banking crises in Table 2 on financial development efficiency shows less return on assets and indicates that banks cannot perform efficiently. However, weaker and inefficient banks may be forced to exit the market during a crisis.

Table 3 explains the gaps between predicted and actual levels of financial development depth, stability, and efficiency. The results indicate that banking efficiency negatively and significantly influences the depth and efficiency gaps while positively influencing the non-performing loans. It implies that greater competitiveness in banking is an optimistic indicator of reducing the depth, stability, and efficiency gaps. Property rights negatively and significantly affect the depth and efficiency gaps, and the connection between non-preforming loans (stability gap) and property rights is positive but statistically insignificant. Similarly, government integrity and financial liberalization also adversely affect the depth, stability, and efficiency gaps. However, the significance level of financial liberalization is low. We conclude that institutions in terms of government integrity, property rights, and financial liberalization considerably matter in lowering the depth, stability, and efficiency gaps and improving the financial sector’s abilities. However, growth increases the gaps as its impact on financial development depth, stability, and efficiency gaps are positive. Hartwell et al. [65] also found similar results in their conclusion on the Russian economy. Banking crises positively impact financial development depth and efficiency gaps while negatively influencing non-performing loans (stability gap). It implies that banking crises amplify the financial development gaps in all dimensions. This study also suggests that foreign direct investment is a growth-enhancing factor as it positively lowers the financial gaps and activates the countries’ economic systems.

**Table 3:** Financial development gap-analysis.

Variables	FD depth Gap (PC) (lag-1)	FD stability Gap (NPLS) (lag-1)	FD efficiency Gap (ROA) (lag -1)	BE	PR	LR	GI	GDP	FDI	BCR_ dummy
FD depth Gap (PC)	0.0076			-0.0218**	-0.0002**	-0.0014	-0.00015	0.050***	-0.00046*	0.0022
	(0.944)			(0.021)	(0.047)	(0.180)	(0.636)	(0.001)	(0.083)	(0.804)
FD stability Gap (NPLS)		0.563***		0.419***	0.0037	0.0049	0.0084***	0.840***	0.0011	-2.089***
		(0.000)		(0.000)	(0.574)	(0.240)	(0.002)	(0.000)	(0.561)	(0.000)
FD efficiency Gap (ROA)			0.171***	-0.285**	-0.0035***	-0.010***	-0.0057***	2.914***	-0.0043**	3.373***
			(0.000)	(0.028)	(0.000)	(0.001)	(0.000)	(0.000)	(0.012)	(0.000)
j-stat	30.917	27.037	29.891							
Prob	(0.521)	(0.715)	(0.573)							
AR(2)	-0.381	-0.134	-0.977							
	(0.702)	(0.8931)	(0.328)							

Note: ***, **, * show significance level at 1%, 5%, and 10% respectively. P-values are given in parenthesis. J-stat prob. greater than 5% shows that instruments are valid

Based on the findings, it is clear from this analysis that the financial sector is significantly important in achieving BRI policy goals and even creates new drivers for development. The president of China also highlighted at the 19^th^ Party Congress that they should promote technological innovation and modern finance to develop effective market mechanisms. They have found that when banks are efficient, the depth of financial development in terms of credits increases. It implies that low lending interest rates would decrease lending risk and increase credit rationing. Suppose the BRI country’s banks are efficient. In that case, they can sagaciously limit all operational risk and motivate savors to invest in growth-stimulating projects by offering them a reasonable and well compensation rate to the scale of the incurred risks. So, the efficiency of BRI states’ banks can shrink the financing gap in BRI projects by mobilizing saving from savors and allocating it to more productive tasks that benefit the financiers and the development of BRI.

Along with banking efficiency, the study found evidence that the other institutional factors have a progressive attitude toward financial development that eventually boost growth in BRI economies. Firstly, property rights can be a source to bring efficient outcomes regarding BRI. A secured property rights system leads them to fully account for all the benefits and costs of engaging their resources in a project. Conversely, although financial liberalization can destabilize the financial system, it is a prerequisite for successful financial development in BRI. It implies that the liberalization of financial entities allows interest rates to reach their competitive market equilibrium, increasing savings, investments, and growth. Additionally, government institutions, in terms of government integrity, can help to promote development through lower costs and increased access to finance.

## 7. Conclusions

The financial sector serves as the primary reservoir through which the Belt and Road Initiative (BRI) secures resources and capital to channel into diverse sectors. Indeed, the banking sector is a potent mechanism that aggregates savings and facilitates investment, fostering economic expansion. Consequently, this study meticulously scrutinized the impacts of banking efficiency, institutional frameworks, and financial development across three pivotal dimensions—depth, stability, and efficiency—from 2007 to 2018. Subsequently, the technical efficacy of the banking sector was quantified through the application of Data Envelopment Analysis (DEA) for BRI-affiliated nations. These efficiency scores, forming a fundamental prerequisite, underpinned the empirical analysis. In the subsequent estimation phase, the Generalized Method of Moments (GMM) approach was employed to dissect the ramifications of banking efficiency on financial development. Recognizing the diversity inherent in financial systems, a solitary indicator as a proxy for financial development would be unduly constricting. Hence, this study harnessed three facets of financial systems—depth, stability, and efficiency—to comprehensively gauge financial development. This multi-dimensional approach stands as an innovative augmentation to existing scholarship.

In tandem, the assessment incorporated benchmarks to discern the financial gaps—encompassing depth, stability, and efficiency—within the cohort of BRI countries. Three salient institutions relevant to the financial sector—financial liberalization, property rights, and government integrity—were embraced. Furthermore, the role of foreign direct investment, a factor frequently overlooked in financial development evaluations, was integrated. The dissection of financial development and its gap analysis incontrovertibly underlines the preeminent role of banking efficiency in shaping financial development’s depth, stability, and efficiency. Other policy variables, inclusive of growth and foreign direct investment, function as catalysts for the sustainable evolution of BRI’s financial architecture. Moreover, robust institutional frameworks corroborate the three-dimensional advancement of the BRI’s financial development domain.

The investigation divulged that the integrity of governance within the BRI context is an indispensable bulwark for securing financial activities. Although the literature’s portrayal of the influence of financial liberalization remains equivocal, this study ascertained a positive linkage between financial liberalization and the expansion of the BRI financial sphere. However, caution is warranted, as financial liberalization may be detrimental to financial stability, despite its positive influence on enhancing development depth and efficiency. Property rights exhibit a favorable alignment across all BRI’s financial sector dimensions. The role of structural variables exerts a constructive impact on bridging the financial depth gap, albeit with modest significance. Conversely, a constructive connection is not discerned between the stability of financial development and the structural variable. Based on these findings, this study proffers recommendations within the context of the BRI financial landscape. Foremost, the study underscores the pivotal role of banking efficiency and advocates for introducing novel products and technologies to deepen the financial sector, thereby fostering growth within BRI member states. Furthermore, the study highlights government integrity as a stabilizing force within the BRI’s financial system, emphasizing the need for reform-minded policies to bolster financial development and engendering confidence among market participants. Moreover, by leveraging property rights policies, governments can bolster the financial trajectory and propel sustainable advancement within BRI nations. Lastly, the potent influence of foreign direct investment emerges as a driver of stability and productivity within financial development. Thus, the study recommends that governments collaboratively strategize to invest in BRI nations, invigorating the financial sector and advancing mutual growth.

## Supporting information

S1 Appendix (DOCX)
