# Supplementary material for: Evaluating the role of banking efficiency, institutions and financial development for sustainable development: Implications for Belt and Road Initiative (BRI)
Source: PLoS One. 2023 Oct 12;18(10):e0290780. doi: 10.1371/journal.pone.0290780 (PMC10569618; doi:10.1371/journal.pone.0290780)
Supplement: S1 Appendix — (DOCX) [file pone.0290780.s001.docx]

**Appendix**

**Table A1:** Country List

| Albania | Estonia | Lithuania | Singapore |
| --- | --- | --- | --- |
| Armenia | Georgia | Malaysia | Slovakia |
| Bangladesh | Hungary | Moldova | Slovenia |
| Belarus | Indonesia | Nepal | Sri Lanka |
| Bosnia and Herzegovina | Israel | Pakistan | Tajikistan |
| Bulgaria | Kazakhstan | Philippines | Thailand |
| Cambodia | Kuwait | Poland | Turkey |
| China | Kyrgyz Republic | Romania | Ukraine |
| Croatia | Latvia | Russia | United Arab Emirates |
| Czech Republic | Lebanon | Saudi Arabia | Vietnam |

**TableA2:** BRI's financial development panel correlation matrix

|  | **FD depth (PC)** | **BE** | **PR** | **FL** | **GI** | **GDP** | **FDI** | **BCD _ dummy** | **pop** | **Age_dep** | **Pop _ den** |
| --- | --- | --- | --- | --- | --- | --- | --- | --- | --- | --- | --- |
| **FD depth (PC)** | 1 |  |  |  |  |  |  |  |  |  |  |
| **BE** | 0.138 | 1 |  |  |  |  |  |  |  |  |  |
| **PR** | 0.262 | 0.133 | 1 |  |  |  |  |  |  |  |  |
| **FL** | 0.154 | 0.020 | 0.573 | 1 |  |  |  |  |  |  |  |
| **GI** | 0.420 | 0.062 | 0.833 | 0.504 | 1 |  |  |  |  |  |  |
| **GDP** | 0.436 | 0.154 | 0.672 | 0.490 | 0.772 | 1 |  |  |  |  |  |
| **FDI** | -0.054 | -0.126 | 0.045 | 0.098 | 0.014 | 0.082 | 1 |  |  |  |  |
| **BCD_dummy** | -0.051 | -0.072 | 0.046 | -0.050 | 0.022 | 0.030 | -0.051 | 1 |  |  |  |
| **POP** | 0.094 | 0.017 | -0.376 | -0.523 | -0.365 | -0.336 | -0.152 | -0.084 | 1 |  |  |
| **Age_dep** | -0.292 | -0.090 | 0.021 | 0.097 | -0.162 | -0.188 | 0.008 | -0.084 | 0.039 | 1 |  |
| **POP_den** | 0.153 | -0.213 | 0.158 | -0.027 | 0.211 | -0.074 | -0.152 | -0.035 | 0.105 | -0.112 | 1 |
|  | **FD stability (NPLS)** | **BE** | **PR** | **FL** | **GI** | **GDP** | **FDI** | **BCD_dummy** | **pop** | **Age_dep** | **Pop _ den** |
| **FD Stability (NPLS)** | 1 |  |  |  |  |  |  |  |  |  |  |
| **BE** | -0.009 | 1 |  |  |  |  |  |  |  |  |  |
| **PR** | -0.220 | 0.132 | 1 |  |  |  |  |  |  |  |  |
| **FL** | 0.052 | 0.025 | 0.576 | 1 |  |  |  |  |  |  |  |
| **GI** | -0.263 | 0.064 | 0.834 | 0.504 | 1 |  |  |  |  |  |  |
| **GDP** | -0.129 | 0.162 | 0.677 | 0.486 | 0.773 | 1 |  |  |  |  |  |
| **FDI** | -0.072 | -0.123 | 0.045 | 0.096 | 0.012 | 0.078 | 1 |  |  |  |  |
| **BCD_dummy** | 0.274 | -0.070 | 0.046 | -0.052 | 0.020 | 0.026 | -0.052 | 1 |  |  |  |
| **POP** | -0.228 | 0.012 | -0.379 | -0.528 | -0.364 | -0.335 | -0.149 | -0.082 | 1 |  |  |
| **Age_dep** | 0.077 | -0.087 | 0.022 | 0.092 | -0.168 | -0.200 | 0.004 | -0.086 | 0.038 | 1 |  |
| **POP_den** | -0.205 | -0.216 | 0.160 | -0.024 | 0.215 | -0.069 | -0.151 | -0.033 | 0.100 | -0.117 | 1 |
|  | **FD efficiency (ROA)** | **BE** | **PR** | **FL** | **GI** | **GDP** | **FDI** | **BCD_dummy** | **pop** | **Age_dep** | **Pop _ den** |
| **FD Efficiency** | 1 |  |  |  |  |  |  |  |  |  |  |
| **(ROA)** |  |  |  |  |  |  |  |  |  |  |  |
| **BE** | 0.068 | 1 |  |  |  |  |  |  |  |  |  |
| **PR** | 0.035 | 0.148 | 1 |  |  |  |  |  |  |  |  |
| **FL** | -0.007 | 0.034 | 0.564 | 1 |  |  |  |  |  |  |  |
| **GI** | 0.122 | 0.050 | 0.835 | 0.509 | 1 |  |  |  |  |  |  |
| **GDP** | 0.043 | 0.151 | 0.670 | 0.493 | 0.762 | 1 |  |  |  |  |  |
| **FDI** | 0.080 | -0.114 | 0.048 | 0.110 | 0.024 | 0.095 | 1 |  |  |  |  |
| **BCD_dummy** | -0.022 | -0.065 | 0.041 | -0.072 | 0.030 | 0.027 | -0.038 | 1 |  |  |  |
| **POP** | 0.020 | 0.026 | -0.386 | -0.534 | -0.383 | -0.369 | -0.167 | -0.088 | 1 |  |  |
| **Age_dep** | 0.092 | -0.099 | 0.012 | 0.096 | -0.184 | -0.213 | 0.028 | -0.091 | 0.029 | 1 |  |
| **POP_den** | -0.111 | -0.186 | 0.185 | -0.020 | 0.245 | -0.044 | -0.158 | -0.028 | 0.111 | -0.114 | 1 |

**TableA3:** BRI's financial development Gap panel correlation matrix

|  | **FD (PC) depth Gap** | **BE** | **PR** | **FL** | **GI** | **GDP** | **FDI** | **BCD_dummy** |
| --- | --- | --- | --- | --- | --- | --- | --- | --- |
| FD (PC) depth Gap | 1 |  |  |  |  |  |  |  |
| BE | -0.035 | 1 |  |  |  |  |  |  |
| PR | 0.013 | 0.133 | 1 |  |  |  |  |  |
| FL | 0.003 | 0.020 | 0.573 | 1 |  |  |  |  |
| GI | 0.005 | 0.062 | 0.833 | 0.504 | 1 |  |  |  |
| GDP | 0.000 | 0.154 | 0.672 | 0.490 | 0.772 | 1 |  |  |
| FDI | 0.040 | -0.126 | 0.045 | 0.098 | 0.014 | 0.082 | 1 |  |
| BCD_dummy | 0.038 | 0.036 | 0.002 | -0.088 | -0.022 | 0.016 | -0.037 | 1 |
|  | **FD Stability Gap _(NPLS)** | **BE** | **PR** | **FL** | **GI** | **GDP** | **FDI** | **BCD_dummy** |
| FD Stability Gap _(NPLS) | 1 |  |  |  |  |  |  |  |
| BE | 0.005 | 1 |  |  |  |  |  |  |
| PR | 0.055 | 0.133 | 1 |  |  |  |  |  |
| FL | 0.017 | 0.020 | 0.573 | 1 |  |  |  |  |
| GI | 0.085 | 0.062 | 0.833 | 0.504 | 1 |  |  |  |
| GDP | 0.005 | 0.154 | 0.672 | 0.490 | 0.772 | 1 |  |  |
| FDI | 0.042 | -0.126 | 0.045 | 0.098 | 0.014 | 0.082 | 1 |  |
| BCD_dummy | 0.362 | 0.036 | 0.002 | -0.088 | -0.022 | 0.016 | -0.037 | 1 |
|  | **FD Efficiency Gap (ROA)** | **BE** | **PR** | **FL** | **GI** | **GDP** | **FDI** | **BCD_dummy** |
| FD Efficiency Gap (ROA) | 1 |  |  |  |  |  |  |  |
| BE | -0.084 | 1 |  |  |  |  |  |  |
| PR | 0.004 | 0.133 | 1 |  |  |  |  |  |
| FL | -0.022 | 0.020 | 0.573 | 1 |  |  |  |  |
| GI | -0.092 | 0.062 | 0.833 | 0.504 | 1 |  |  |  |
| GDP | -0.085 | 0.154 | 0.672 | 0.490 | 0.772 | 1 |  |  |
| FDI | 0.003 | -0.126 | 0.045 | 0.098 | 0.014 | 0.082 | 1 |  |
| BCD_dummy | -0.001 | 0.036 | 0.002 | -0.088 | -0.022 | 0.016 | -0.037 | 1 |

**Table A4.** Efficiency Score of BRI Economies Banks

| **countries** | **2007** | **2008** | **2009** | **2010** | **2011** | **2012** | **2013** | **2014** | **2015** | **2016** | **2017** | **2018** | **Average** |
| --- | --- | --- | --- | --- | --- | --- | --- | --- | --- | --- | --- | --- | --- |
| Albania | 0.1494 | 0.1594 | 0.1794 | 0.2094 | 0.2494 | 0.2894 | 0.3888 | 0.3588 | 0.1914 | 0.3487 | 0.3794 | 0.3203 | 0.2686 |
| Armenia | 0.0223 | 0.0323 | 0.0523 | 0.0823 | 0.1223 | 0.1723 | 0.3332 | 0.3174 | 0.125 | 0.2169 | 0.2749 | 0.3025 | 0.1711 |
| Bangladesh | 0.0661 | 0.0961 | 0.1261 | 0.1561 | 0.1961 | 0.2461 | 0.2884 | 0.2721 | 0.1512 | 0.1867 | 0.1855 | 0.2761 | 0.1872 |
| Belarus | 0.0756 | 0.0856 | 0.1056 | 0.1356 | 0.1756 | 0.2256 | 0.2586 | 0.2537 | 0.2081 | 0.2613 | 0.2507 | 0.2429 | 0.1899 |
| Bosnia and Herzegovina | 0.0673 | 0.0773 | 0.0973 | 0.1273 | 0.1673 | 0.2173 | 0.2404 | 0.2199 | 0.1248 | 0.2087 | 0.2388 | 0.248 | 0.1695 |
| Bulgaria | 0.1014 | 0.1114 | 0.1314 | 0.1614 | 0.2014 | 0.2514 | 0.2886 | 0.275 | 0.1675 | 0.2798 | 0.3094 | 0.3707 | 0.2207 |
| Cambodia | 0.1586 | 0.1686 | 0.1886 | 0.2186 | 0.2586 | 0.3086 | 0.4387 | 0.4139 | 0.2363 | 0.3854 | 0.4254 | 0.3933 | 0.2995 |
| China | 0.0717 | 0.0817 | 0.1017 | 0.1317 | 0.1717 | 0.2217 | 0.4462 | 0.4061 | 0.1951 | 0.2914 | 0.3358 | 0.397 | 0.237 |
| Croatia | 0.0098 | 0.0098 | 0.0298 | 0.0598 | 0.0998 | 0.1498 | 0.2264 | 0.2278 | 0.1066 | 0.2058 | 0.2418 | 0.2483 | 0.134 |
| Czech Republic | 0.1739 | 0.1839 | 0.2039 | 0.2339 | 0.2739 | 0.3239 | 0.4055 | 0.4254 | 0.1902 | 0.307 | 0.3644 | 0.3673 | 0.2877 |
| Estonia | 0.1691 | 0.1791 | 0.1991 | 0.2291 | 0.2691 | 0.3191 | 0.3286 | 0.3361 | 0.1886 | 0.2977 | 0.3476 | 0.3477 | 0.2675 |
| Georgia | 0.0342 | 0.0442 | 0.0642 | 0.0942 | 0.1342 | 0.1842 | 0.3085 | 0.2996 | 0.2488 | 0.285 | 0.3026 | 0.2776 | 0.1897 |
| Hungary | 0.01 | 0.02 | 0.03 | 0.04 | 0.1206 | 0.1706 | 0.198 | 0.1703 | 0.1056 | 0.1583 | 0.186 | 0.2574 | 0.1222 |
| Indonesia | 0.0406 | 0.0506 | 0.0706 | 0.1006 | 0.1406 | 0.1906 | 0.356 | 0.3035 | 0.1607 | 0.2543 | 0.283 | 0.2788 | 0.1858 |
| Israel | 0.0337 | 0.0437 | 0.0637 | 0.0937 | 0.1337 | 0.1837 | 0.2274 | 0.2307 | 0.1455 | 0.2215 | 0.253 | 0.2524 | 0.1568 |
| Kazakhstan | 0.2566 | 0.2666 | 0.2866 | 0.3166 | 0.3566 | 0.4066 | 0.4961 | 0.4568 | 0.3836 | 0.3796 | 0.4457 | 0.4572 | 0.3757 |
| Kuwait | 0.1886 | 0.1986 | 0.2186 | 0.2486 | 0.2886 | 0.3386 | 0.5093 | 0.4513 | 0.2402 | 0.3443 | 0.3749 | 0.3784 | 0.315 |
| Kyrgyz Republic | 0.1002 | 0.1102 | 0.1302 | 0.1602 | 0.2002 | 0.2502 | 0.3395 | 0.2511 | 0.124 | 0.1642 | 0.1975 | 0.2048 | 0.1860 |
| Latvia | 0.1226 | 0.1326 | 0.1526 | 0.1826 | 0.2226 | 0.2726 | 0.2528 | 0.2868 | 0.163 | 0.2258 | 0.2367 | 0.2861 | 0.2114 |
| Lebanon | 0.0489 | 0.0589 | 0.0789 | 0.1089 | 0.1489 | 0.1989 | 0.2997 | 0.2921 | 0.1379 | 0.2058 | 0.2252 | 0.2753 | 0.1732 |
| Lithuania | 0.085 | 0.095 | 0.115 | 0.145 | 0.185 | 0.235 | 0.2818 | 0.3104 | 0.1811 | 0.2711 | 0.3127 | 0.3609 | 0.2148 |
| Malaysia | 0.0897 | 0.0997 | 0.1197 | 0.1497 | 0.1897 | 0.2397 | 0.311 | 0.3001 | 0.1634 | 0.2265 | 0.2507 | 0.3385 | 0.2065 |
| Moldova | 0.0622 | 0.0722 | 0.0922 | 0.1222 | 0.1622 | 0.2122 | 0.2455 | 0.2592 | 0.1741 | 0.2325 | 0.2165 | 0.2365 | 0.173 |
| Nepal | 0.0537 | 0.0637 | 0.0837 | 0.1137 | 0.1537 | 0.2037 | 0.4304 | 0.3814 | 0.1833 | 0.3173 | 0.3782 | 0.3722 | 0.2279 |
| Pakistan | 0.0058 | 0.0158 | 0.0358 | 0.0658 | 0.1058 | 0.1558 | 0.241 | 0.2935 | 0.1485 | 0.2036 | 0.2265 | 0.2353 | 0.1444 |
| Philippines | 0.1012 | 0.1112 | 0.1312 | 0.1612 | 0.2012 | 0.2512 | 0.3111 | 0.2889 | 0.1384 | 0.2497 | 0.2872 | 0.2264 | 0.2049 |
| Poland | 0.0713 | 0.0813 | 0.1013 | 0.1313 | 0.1713 | 0.2213 | 0.2544 | 0.2396 | 0.1152 | 0.2128 | 0.2531 | 0.2375 | 0.1742 |
| Romania | 0.0435 | 0.0535 | 0.0735 | 0.1035 | 0.1435 | 0.1935 | 0.2503 | 0.2258 | 0.1172 | 0.2079 | 0.2781 | 0.3055 | 0.1663 |
| Russia | 0.37 | 0.38 | 0.4 | 0.43 | 0.47 | 0.52 | 0.5019 | 0.4774 | 0.2328 | 0.3539 | 0.4367 | 0.3933 | 0.4138 |
| Saudi Arabia | 0.0925 | 0.1025 | 0.1225 | 0.1525 | 0.1925 | 0.2425 | 0.322 | 0.3037 | 0.1751 | 0.2354 | 0.2936 | 0.3167 | 0.2126 |
| Singapore | 0.1121 | 0.1221 | 0.1421 | 0.1721 | 0.2121 | 0.2621 | 0.2584 | 0.3591 | 0.1684 | 0.27 | 0.2878 | 0.3601 | 0.2272 |
| Slovakia | 0.128 | 0.138 | 0.158 | 0.188 | 0.228 | 0.278 | 0.3361 | 0.3165 | 0.16 | 0.2798 | 0.3103 | 0.3107 | 0.2359 |
| Slovenia | 0.112 | 0.1220 | 0.1420 | 0.17206 | 0.21206 | 0.26206 | 0.2584 | 0.35906 | 0.1684 | 0.26996 | 0.28778 | 0.36008 | 0.2271 |
| Sri Lanka | 0.0121 | 0.0221 | 0.0421 | 0.0721 | 0.1121 | 0.1621 | 0.3064 | 0.2837 | 0.1357 | 0.2041 | 0.2409 | 0.2457 | 0.15325 |
| Tajikistan | 0.169 | 0.179 | 0.199 | 0.229 | 0.269 | 0.319 | 0.3061 | 0.3519 | 0.3014 | 0.2097 | 0.3428 | 0.3634 | 0.26994 |
| Thailand | 0.0599 | 0.0699 | 0.0899 | 0.1199 | 0.1599 | 0.2099 | 0.3953 | 0.3539 | 0.1785 | 0.2715 | 0.3122 | 0.3374 | 0.21318 |
| Turkey | 0.0661 | 0.0761 | 0.0961 | 0.1261 | 0.1661 | 0.2161 | 0.334 | 0.3141 | 0.1871 | 0.2677 | 0.2831 | 0.3692 | 0.20848 |
| Ukraine | 0.0381 | 0.0481 | 0.0681 | 0.0981 | 0.1381 | 0.1881 | 0.2642 | 0.2726 | 0.1717 | 0.2256 | 0.2462 | 0.2814 | 0.17002 |
| United Arab Emirates | 0.1747 | 0.1847 | 0.2047 | 0.2347 | 0.2747 | 0.3247 | 0.4213 | 0.424 | 0.2241 | 0.3236 | 0.3511 | 0.3651 | 0.29228 |
| Vietnam | 0.0702 | 0.0802 | 0.1002 | 0.1302 | 0.1702 | 0.2202 | 0.3515 | 0.3372 | 0.1501 | 0.2466 | 0.2996 | 0.3424 | 0.20821 |
